# Efficacy of High-Intensity Focused Ultrasound Combined With GnRH-a for Adenomyosis: A Systematic Review and Meta-Analysis

**DOI:** 10.3389/fpubh.2021.688264

**Published:** 2021-08-16

**Authors:** Li-Li Pang, Jin Mei, Ling-Xiu Fan, Ting-Ting Zhao, Ruo-Nan Li, Yi Wen

**Affiliations:** Hospital of Chengdu University of Traditional Chinese Medicine, Chengdu, China

**Keywords:** high-intensity focused ultrasound, gonadotropin-releasing hormone agonist, GnRH-a, adenomyosis, meta-analysis

## Abstract

**Objective:** High-intensity focused ultrasound (HIFU) is an innovative non-invasive technology used for adenomyosis. Gonadotropin-releasing hormone agonist (GnRH-a) is a hormone commonly used for adenomyosis. We investigated and assessed the efficacy of HIFU combined with GnRH-a for adenomyosis.

**Methods:** For this systematic review and meta-analysis, we searched Pubmed, Cochrane Library, Web of Science, Embase, CNKI, WanFang, and VIP databases for relevant articles published in Chinese or English that compared HIFU combined with GnRH-a vs. HIFU alone in patients with adenomyosis. The last literature search was completed on January 31, 2021. Two reviewers independently assessed study eligibility and assessed risk of bias. Another two reviewers extracted the data. The RevMan5.3 software was used for the data analysis. Changes in volume of the uterine and adenomyotic lesion were defined as the primary outcomes. The secondary outcomes were visual analog scale (VAS) scores for dysmenorrhea, menstrual volume scores, serum CA125 levels, and recurrence rate. This study is registered with PROSPERO (CRD42021234301).

**Results:** Three hundred and ninety potentially relevant articles were screened. Nine studies with data for 766 patients were finally included. Compared with the HIFU alone group, the HIFU combined with GnRH-a group had a higher rate of uterine volume reduction (MD 7.51, 95% CI 5.84–9.17, *p* < 0.00001), smaller adenomyotic lesion volume (MD 4.11, 95% CI 2.93–5.30, *p* < 0.00001), lower VAS score for dysmenorrhea (MD 1.27, 95% CI 0.54–2.01, *p* = 0.0007) and menstrual volume score (MD 0.88, 95% CI 0.73–1.04, *p* < 0.00001), and lower CA125 level (SMD 0.31, 95% CI 0.05–0.56, *p* = 0.02) after the procedure. The recurrence rate in the HIFU combined with GnRH-a group was lower than that in the HIFU alone group (RR 0.28, 95% CI 0.10–0.82, *p* = 0.02).

**Conclusions:** Compared with HIFU treatment alone, HIFU combined with GnRH-a for the treatment of adenomyosis has greater efficacy in decreasing the volumes of the uterine and adenomyotic lesions and alleviating symptoms. However, since the number of the included studies was too small and most of them were written in Chinese, this conclusion needs to be referenced with caution. And the long-term evidence of its efficacy is still insufficient.

**Systematic Review Registration**: https://www.crd.york.ac.uk/prospero/ identifier [CRD42021234].

## Introduction

Adenomyosis is a gynecological disease characterized by ectopic endometrial tissue in the myometrium (1). It often occurs in women aged 30−40 years. The current prevalence of adenomyosis ranges from 20 to 35% (2, 3). The main clinical symptoms of the patients include abnormal uterine bleeding, dysmenorrhea, and infertility. Sex steroid hormone aberrations, inflammation, changes in cell proliferation, and neuroangiogenesis may be the key pathogenic mechanisms (4).

The treatment of adenomyosis includes medication and minimally invasive/surgical treatment (5). Medications include gonadotropin-releasing hormone (GnRH) analogs, progesterone, combined oral contraceptives, and non-steroidal anti-inflammatory drugs. Traditional minimally invasive/surgical treatments include hysterectomy and uterine artery embolization (UAE). However, hysterectomy is not a good choice for women who want to remain fertile. Although UAE treatment can improve patient symptoms, its effects on ovarian function and pregnancy are still uncertain (2).

High-intensity focused ultrasound (HIFU), as an emerging non-invasive technique for the treatment of benign tumors, has been used for adenomyosis since 2008 (6). Under ultrasonography or magnetic resonance (MRI) inspection, HIFU can cause high-intensity ultrasound energy to act on abnormal target tissues and eliminate the lesion through thermal and cavitation effects, and allows for the preservation of normal tissue around the lesion (7). In recent years, HIFU therapy has become a good alternative surgery for patients who want to preserve their uterus (8). However, adenomyosis is an estrogen-dependent disease, and HIFU treatment will not change the hormone status in the body. The risk of recurrence still exists. Gonadotropin-releasing hormone agonist (GnRH-a) is a hormone commonly used for adenomyosis. It can decrease the estrogen level to the menopausal level and promote the atrophy of adenomyotic lesions (9).

We did a systematic review and meta-analysis to investigate and evaluate the efficacy of HIFU combined with GnRH-a for adenomyosis and provide evidence-based medical evidence for the clinical application.

## Methods

### Search Strategy

This meta-analysis is conducted in accordance with the Preferred Reporting Items for Systematic Reviews and Meta-analyses (PRISMA) guidelines and was registered in PROSPERO (CRD42021234301). Two reviewers (LLP and JM) searched relevant studies published in Chinese or English using PubMed, Cochrane Library, Web of Science, Embase, CNKI (China National Knowledge Infrastructure), WanFang, and VIP (China Science and Technology Journal Database) databases from their inception dates to January 31, 2021. The following terms were used to search for all possible publications: “Adenomyosis,” “High-intensity focused ultrasound,” and “Gonadotropin-releasing hormone agonist.” The English search formula is ((Adenomyos^*^) AND ((“Gonadotropin-Releasing Hormone”[Mesh]) OR (GnRH agonist))) OR ((Adenomyos^*^) AND ((“High-intensity focused ultrasound ablation” [Mesh]) OR (HIFU))).

### Selection Criteria

The studies included in this meta-analysis met the following criteria: Vannuccini and Petraglia. (1) studies comparing HIFU combined with GnRH-a vs. HIFU alone in patients with adenomyosis. The HIFU combined with GnRH-a group was defined as experimental group, HIFU alone group was defined as control group. Struble et al. (2) Studies object: (1) women aged 18–50 years; (2) women with focal or diffuse adenomyosis diagnosed using ultrasonography, MRI, or computed tomography (CT); (3) patients who had not received any treatment for adenomyosis within 3 months before the study. Abbott (3) Outcome indicator: main outcome indicators are changes in volume of the uterine and adenomyotic lesions were defined as the primary outcome. Secondary outcomes were visual analog scale (VAS) scores for dysmenorrhea, menstrual volume scores, serum CA125 levels, and recurrence rate.

The exclusion criteria are as follows: (1) Reviews, animal experiments, case reports, conference abstracts, conference proceedings, editorial letters, guidance or comments; (2) repeated studies; (3) studies where full text is not available; (4) patients with uterine fibroids or other gynecological diseases, whose clinical symptoms are similar with those of adenomyosis, were also excluded; (5) studies reporting clinical outcomes at a follow-up shorter than 3 months were excluded because it takes some time for the lesions to absorb themselves after HIFU ablation.

### Quality Assessment

Two investigators (LL P and J M) independently screened titles and abstracts for eligibility as well as the full text of each eligible study, to confirm the inclusion criteria. The quality evaluation criteria of individual studies was assessed using the risk bias assessment tool in the Cochrane Evaluation Manual (10). This assessment tool include selection bias, performance bias, detection bias, attrition bias, reporting bias and other bias.

### Data Extraction

Two authors (RN L and TT Z) were responsible for data extraction, and another two authors (LL P and J M) verified the accuracy of data. Disagreements were solved by consensus. If it cannot be resolved, it was resolved through consultation with experts (Y W). The following data were obtained from the studies: first author, publication year, study design, sample size, average age, imaging tools for the diagnosis of adenomyosis, last follow-up time, total energy and average power during HIFU treatment. The primary efficacy endpoints were changes in volume of uterine and adenomyotic lesions. The secondary outcome indicators were VAS score for dysmenorrhea, menstrual volume scores, serum CA125 levels, and recurrence. Pregnancy outcomes, when available, were also extracted.

### Statistical Analysis

The RevMan5.3 software was used for the data analysis by LX F. Binary variables are expressed as risk ratios (RR), and continuous variables are expressed as mean difference (MD). When the count units are inconsistent, the combined effect size is expressed as a standard mean difference (SMD). The 95% confidence interval (CI) is also expressed. We used the Chi-squared tests and Higgins' *I*^2^ statistics to estimate heterogeneity among studies, with *I*^2^ < 30% indicating low heterogeneity, more than 30% indicating high heterogeneity. The random-effects model was used for high heterogeneity, whereas the fixed-effect model was used for low heterogeneity. *P* < 0.05 were considered significant. If there is a high heterogeneity in studies, a sensitivity analysis will be performed by sequentially removing each single study. Descriptive analyses were used if the data cannot be combined.

## Results

### Selected Study Characteristics

**Figure 1:**
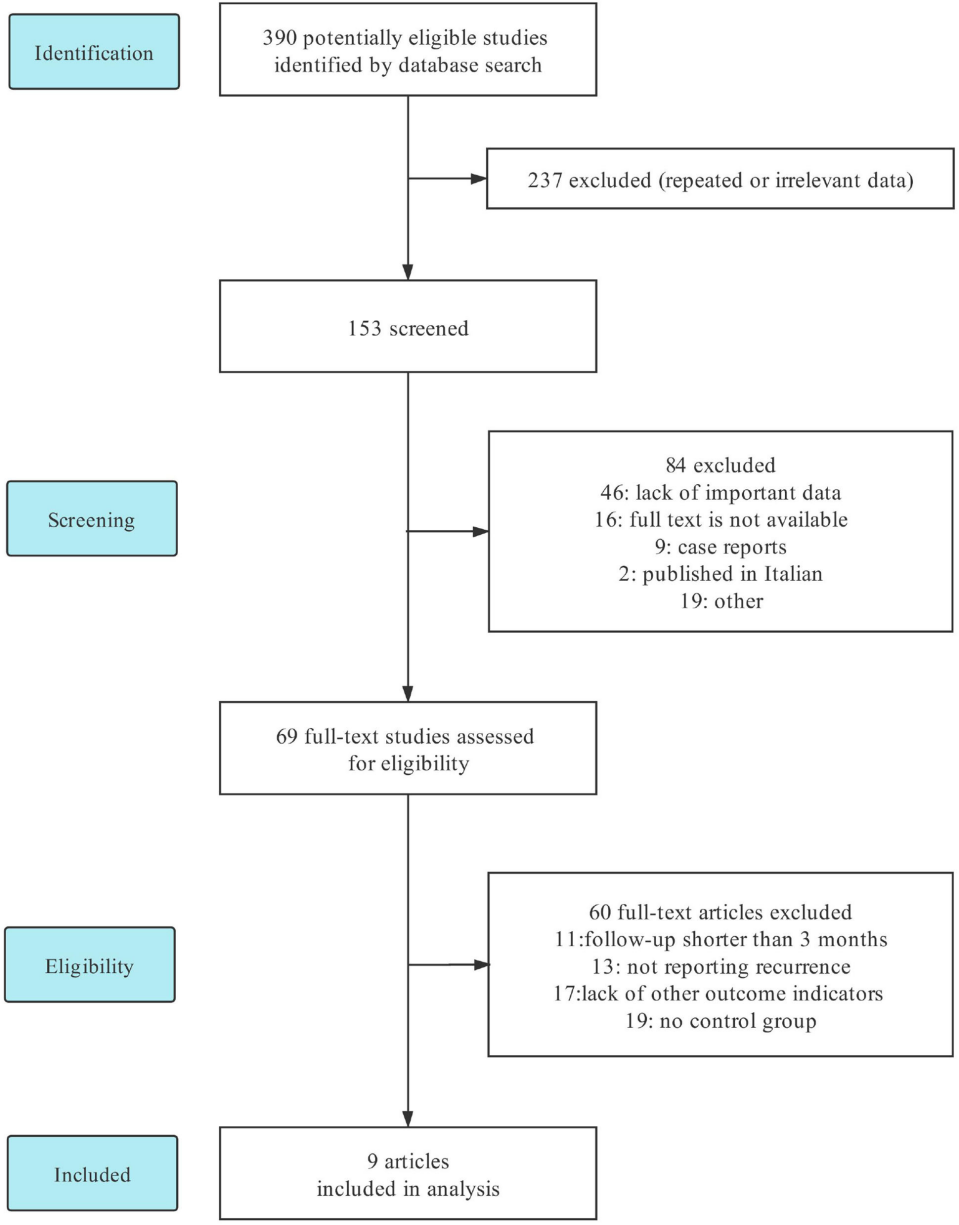
Flow diagram showing selection of articles for this systematic review.

Of the 390 articles collected, 237 articles with duplicate or irrelevant data were excluded. After browsing the title and abstract, 84 were excluded for different reasons (46 articles were lack of important data, 16 full-texts were not available, nine articles were case reports, two articles were published in Italian, and 19 articles were excluded with other reasons). Sixty-nine full-text were assessed eligible, however, 60 were excluded for different reasons (11 articles' follow-up time were shorter than 3 months, 13 articles did not report recurrence, 17 articles were lack of other outcome indicators, and 19 articles had no control group). Nine studies with data for 766 patients were finally included in the present meta-analysis (11–19). The literature screening process is shown in Figure 1.

The experimental group included 346 cases, and the control group included 420 cases. Three were randomized controlled trials (12, 17, 19) and six were clinical controlled trials (11, 13–16, 18). Last follow-up time was 3 months in one study, 6 months in two studies, 12 months in six studies. Three studies mentioned the method of random sequence generation (12, 17, 19). Of the nine studies, one used MRI for the imaging diagnosis of adenomyosis, six used transvaginal ultrasonography or MRI, and three did not report specific imaging diagnostic methods. Although these studies provided information on the imaging diagnostic methods used, they did not provide the specific imaging criteria for the diagnosis of adenomyosis. The baseline characteristics of the included articles is shown in Table 1.

**Table 1:** Characteristics of included studies.

**References**	**Study design**	**Random sequence generation**	**Study group**	**Control group**	**Sample size (n)**	**Age (yeas)**	**Last follow-up time (months)**	**Diagnostic imaging**	**Total energy (J)**	**Average power (W)**
Guo et al. (11)	Prospective	NA	HIFU+GnRH-a	HIFU	24/55	41.00 ± 4.74/39.6 ± 5.3	6	MRI	NA	350~400
Yang and Xie (12)	Retrospective	random number table	HIFU+GnRH-a	HIFU	38/38	41.63 ± 6.36/41.73 ± 6.24	12	NA	NA	NA
Xiao-Ying et al. (13)	Retrospective	NA	HIFU+GnRH-a	HIFU	23/38	41 ± 3.53/41.24 ± 7.07	12	TVUS/MRI	398.26 ± 0/399.08 ± 0.71	392.73 ± 63.64/412.22 ± 315.23
Guo et al. (14)	Retrospective	NA	HIFU+GnRH-a	HIFU	18/45	41.44 ± 4.74/42.42 ± 5.09	12	TVUS/MRI	327793.89 ± 26690.71/303510.29 ± 18634.14	308.11 ± 12.41/272.07 ± 7.34
Li et al. (15)	Retrospective	NA	HIFU+GnRH-a	HIFU	70/64	26-53	12	NA	36900~596200	293~400
Jiang et al. (16)	Prospective	NA	HIFU+GnRH-a	HIFU	45/46	40.96 ± 4.42/39.98 ± 4.22	3	TVUS/MRI	298905.24 ± 24784.77/301886.68 ± 25323.4	294.32 ± 19.07/286.54 ± 13.66
Tan and Li (17)	Retrospective	random number table	HIFU+GnRH-a	HIFU	42/42	38.03 ± 7.21/37.85 ± 7.14	6	TVUS/MRI	NA	NA
Xu et al. (18)	Retrospective	NA	HIFU+GnRH-a	HIFU	41/48	41.9 ± 4.3/43.6 ± 5.1	12	TVUS/MRI	NA	50~400
Yang et al. (19)	Retrospective	random number table	HIFU+GnRH-a	HIFU	40/40	40.23 ± 5.42/40.11 ± 5.52	12	NA	NA	NA

*HIFU, High-Intensity Focused Ultrasound; GnRH-a, Gonadotropin-Releasing Hormone; TVUS, Transvaginal Ultrasound; MRI, Magnetic Resonance Imaging; NA, Not applicable*.

### Quality Assessment

**Figure 2:**
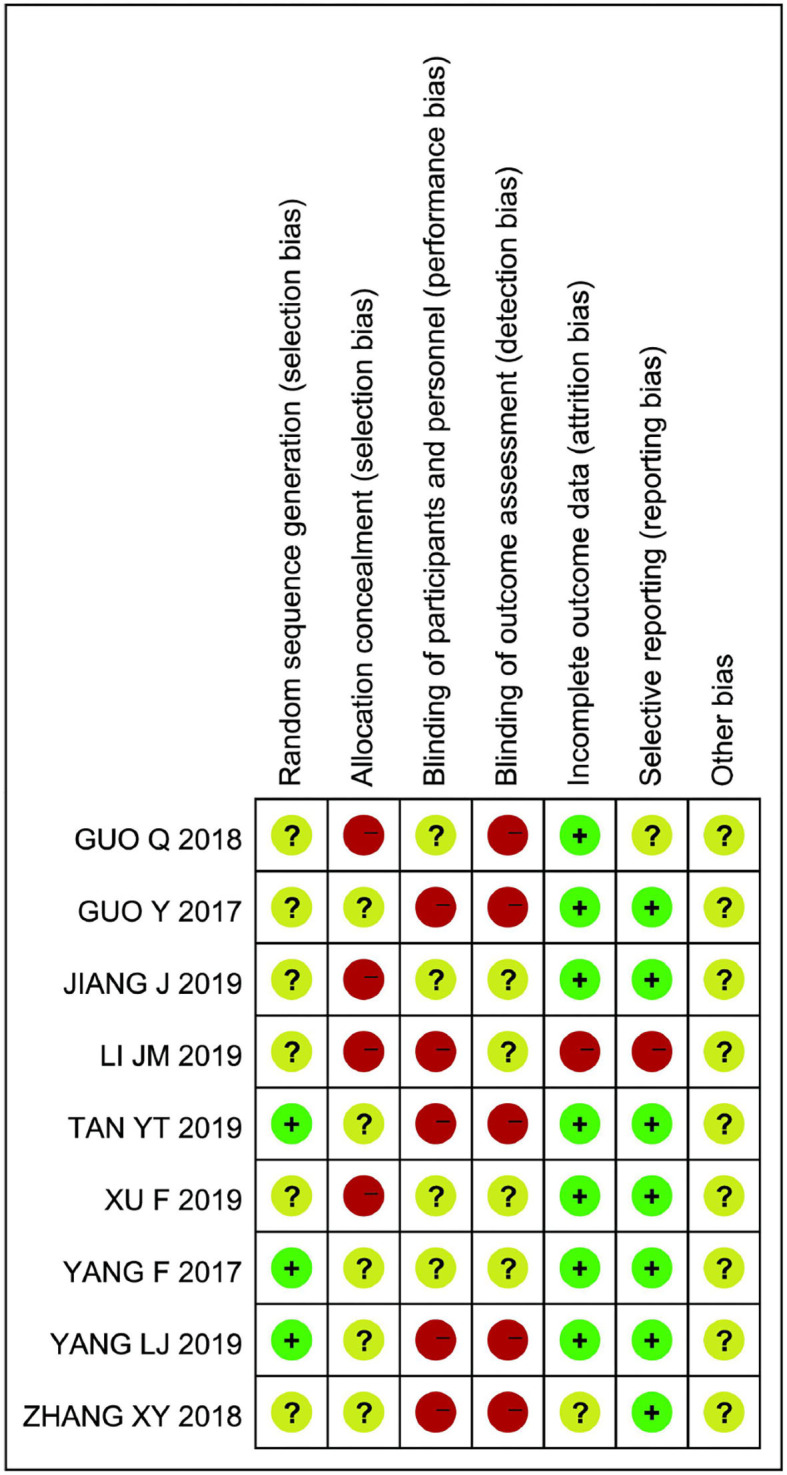
Review authors' judgements about each risk of bias item for each of included study.

**Figure 3:**
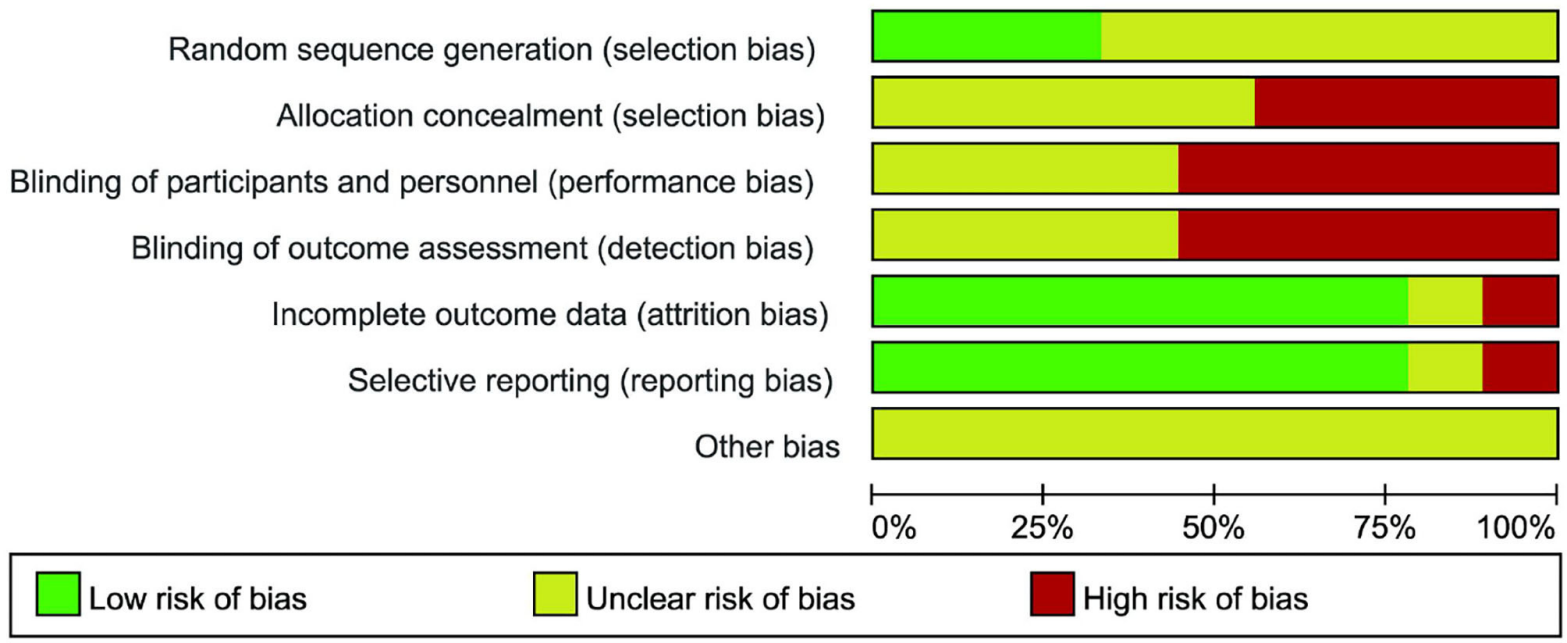
Review authors' judgements about each risk of bias item presented as percentages across all included study.

The quality assessment of the studies is shown in Figure 2. Among the nine included studies, only three (13, 18, 20) reported the method of generating the random allocation sequence, which was the random number table method. All the studies did not mention the random allocation hiding and blinding methods. The specific risk bias analysis is shown in Figure 3.

### Outcome Measures

#### Changes in Uterine Volume

**Figure 4:**
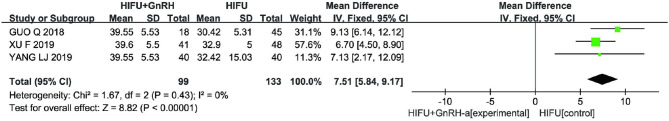
Meta-analysis of uterine volume changes in two groups.

In the three studies included (14, 18, 19), the uterine volume reduction rate after HIFU was evaluated in 232 patients. The meta-analysis results showed that the uterine volume reduction rate in the HIFU with GnRH-a group was higher than that in the HIFU alone group at 12 months after the procedure (MD 7.51, 95% CI 5.84–9.17, *p* < 0.00001, *I*^2^ = 0%; Figure 4).

#### Changes in Adenomyotic Lesion Volume

**Figure 5:**
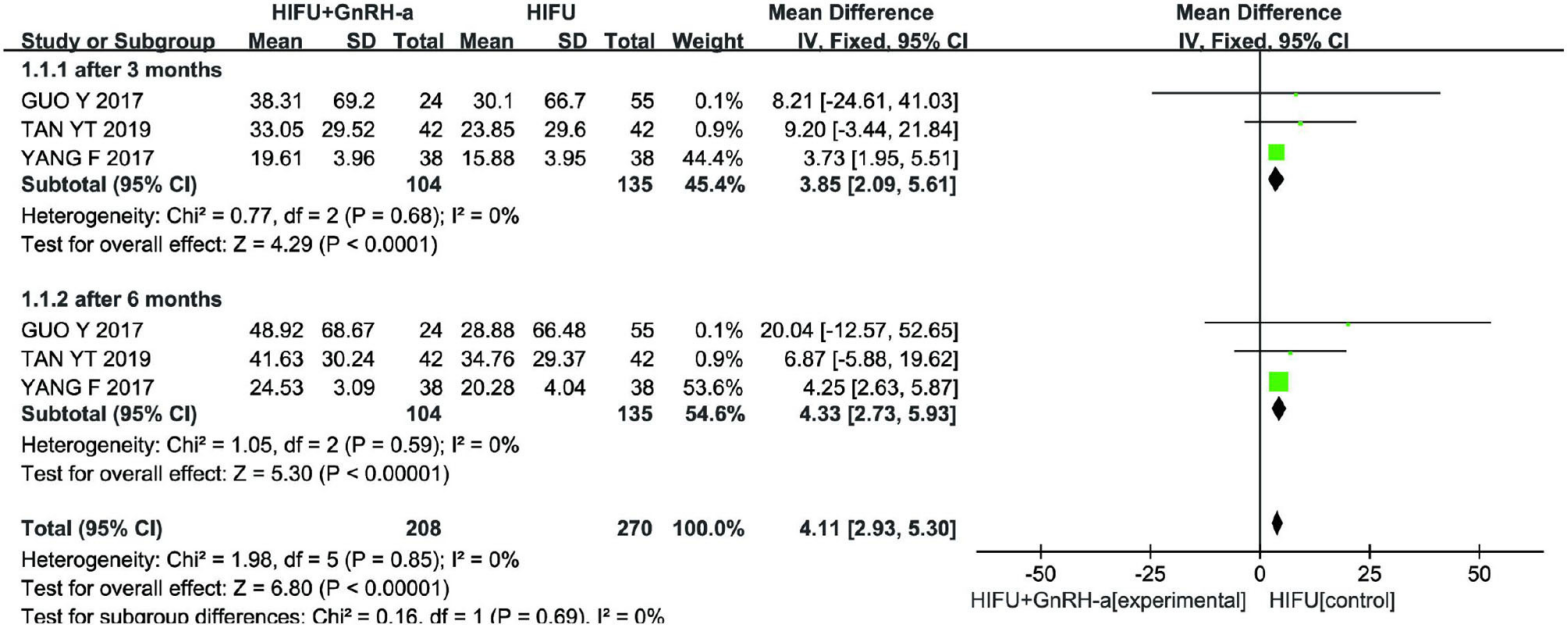
Meta-analysis of lesion volume changes in two groups.

Three studies (11, 12, 17) (239 cases) reported changes in lesion size before and after treatment. The meta-analysis results showed that the lesion volume in the experimental group was smaller than that in the control group (MD 4.11, 95% CI 2.93–5.30, *p* < 0.00001, *I*^2^ = 0%; Figure 5) at 3 and 6 months after the procedure. According to the follow-up time, we divided the articles into two subgroups with a follow-up of 3 months (MD 3.85, 95% CI 2.09–5.61, *p* < 0.00001, *I*^2^ = 0%) and a follow-up of 6 months (MD 4.33, 95% CI 2.73–5.93, *p* < 0.00001, *I*^2^ = 0%), and conducted a subgroup analysis. The results showed that there was no significant difference in the two groups (*p* > 0.05).

#### VAS Scores for Dysmenorrhea

**Figure 6:**
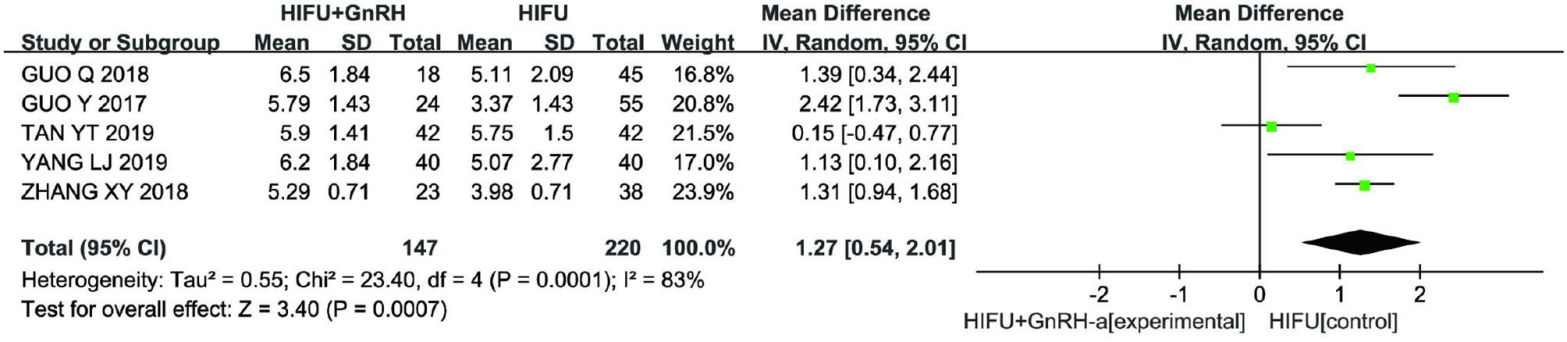
Meta-analysis of visual analog scale (VAS) scores of dysmenorrhea in two groups.

**Figure 7:**
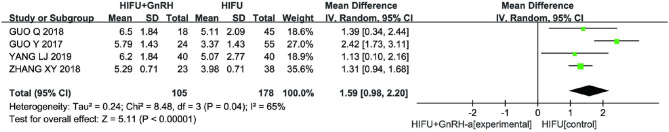
Sensitivity analysis of visual analog scale (VAS) scores of dysmenorrhea in two groups.

A total of five studies (11, 13, 14, 17, 18) (367 cases) used the VAS to assess the dysmenorrhea of patients. The meta-analysis results showed that the VAS scores for dysmenorrhea in the HIFU with GnRH-a group was lower than that in the HIFU alone group after the procedure (MD 1.27, 95% CI 0.54–2.01, *p* = 0.0007, *I*^2^ = 83%; Figure 6). However, the heterogeneity analysis shows an high heterogeneity. Therefore, we performed a sensitivity analysis by sequentially removing each single study. When the TAN 2019 study was removed, the heterogeneity is reduced (MD 1.59, 95% CI 0.98–2.20, *p* < 0.00001, *I*^2^ = 65%; Figure 7).

#### Menstrual Volume Scores

**Figure 8:**
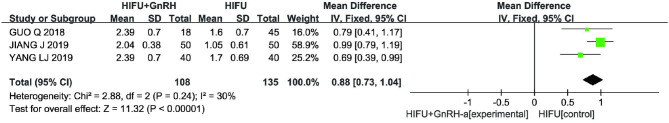
Meta-analysis of menstrual volume scores in two groups.

Three studies (14, 16, 19) (243 cases) used the menstrual volume scores to assess menstrual bleeding. The meta-analysis results showed that the menstrual volume score of the HIFU with the GnRH-a group was lower than that of the HIFU alone group after the procedure (MD 0.88, 95% CI 0.73–1.04, *p* < 0.00001, *I*^2^ = 30%; Figure 8).

#### Serum CA125 Levels

**Figure 9:**
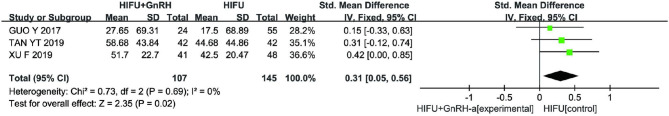
Meta-analysis of serum CA-125 levels in two groups.

Three studies (11, 17, 18) (252 cases) evaluated the serum CA125 levels of the patients. The meta-analysis results showed that the serum CA125 level of the HIFU with GnRH-a group was lower than that of the HIFU alone group after the procedure (SMD 0.31, 95% CI 0.05–0.56, *p* = 0.02, *I*^2^ = 0%; Figure 9).

#### Recurrence Rate

**Figure 10:**
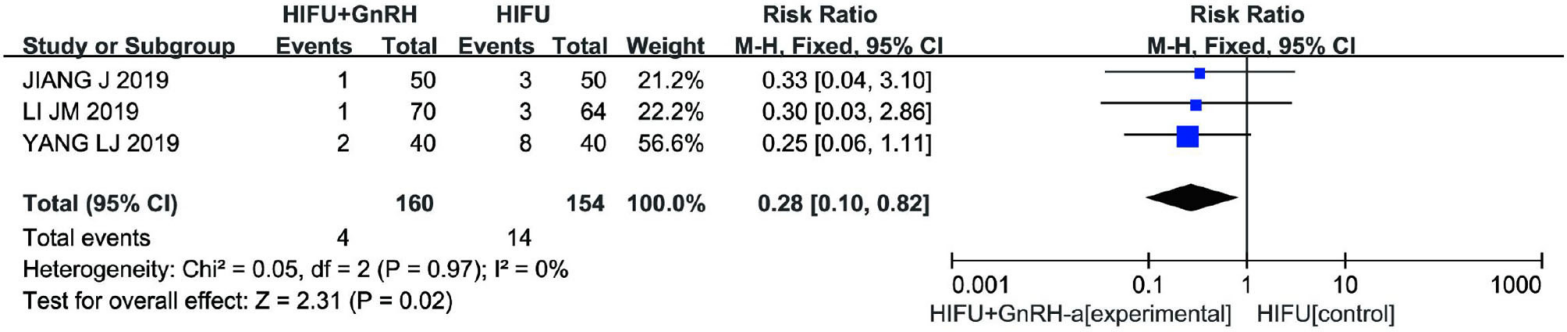
Meta-analysis of recurrence rate in two groups.

Three studies (15, 16, 19) (314 cases) compared the recurrence rates of the experimental and control groups. The meta-analysis results showed that the recurrence rate in the HIFU with GnRH-a group was lower than that in the HIFU alone group (RR 0.28, 95% CI 0.10–0.82, *p* = 0.02, *I*^2^ = 0%; Figure 10).

#### Pregnancy Outcomes

One study reported pregnancy outcomes of the patients at 6 months after treatment. There were five pregnancies reported after intervention in HIFU combined with GnRH-a (*n* = 45), of which three resulted in natural childbirth and two ended in an abortion. In HIFU alone group (*n* = 46), there were four pregnancies reported after HIFU ablation, of which one resulted in natural childbirth, one resulted in miscarriage and two ended in abortion.

## Discussion

The present study is the first systematic review and meta-analysis investigating HIFU combined with GnRH-a for adenomyosis. The results of this meta-analysis of data from 766 patients showed that, compared with HIFU alone, HIFU combined with GnRH-a for the treatment of adenomyosis has greater efficacy in decreasing the volumes of the uterine and adenomyotic lesions and alleviating symptoms.

Adenomyosis is a common and difficult gynecological disease that seriously affects women's health and quality of life. Effective symptom relief, prevention of recurrence, and increasing the pregnancy rate are issues that should be solved. Compared to traditional treatment, HIFU is an non-invasive and innovative technology for adenomyosis (21). However, there is still at risk of recurrence. Therefore, HIFU combined with other therapies for adenomyosis has become the hotspot and trend in research in recent years. HIFU combined with GnRH-a is one of these methods, but no systematic review has been conducted.

The therapeutic mechanism of HIFU is to focus the ultrasound beams emitted outside the body on the targeted lesion. The thermal and cavitation effects converted by mechanical effect of ultrasound cause the temperature of the target tissue at the focal point raise above 60–100°C, in order to cause non-coagulable necrosis of the lesion. At the same time, the surrounding structures are not be damaged (22). Previous studies found that uterine smooth muscle tissue in adenomyotic lesions is sensitive to HIFU treatment (20). HIFU is an effective and ideal treatment for adenomyosis. A retrospective study by Lee et al. enrolled 889 patients with adenomyosis who underwent ultrasound-guided HIFU (USgHIFU). The results revealed that the reduction rates of uterine volume were 44.5, 50.7, and 60.1% at 3, 6, and 12 months after procedure, respectively (23). This is consistent with the results of a recent systematic and meta-analysis which showed a great effect in reducing the uterine volume after HIFU treatment for adenomyosis at 12 months (SMD: 0.85) (24).

GnRH-a is a synthetic derivative of the gonadotropin-releasing hormone. It was first reported in 1991 for the treatment of adenomyosis and obtained good outcomes (25). GnRH-a can combine tightly with the GnRH receptors of the pituitary, so it can competitively downregulate the GnRH receptors in the body, thereby decreasing the level of gonadotropins secreted by the pituitary gland (26). The excitability of ovarian function is also decreased because of this, which inhibits the secretion of estrogen, promotes intimal atrophy, and prevents the continued expansion of the lesion. Up to now, many studies have confirmed the efficacy and safety of GnRH-a. Previous studies reported that GnRH-a can effectively decrease the uterine volume of patients with adenomyosis (27). To evaluate the improvement in chronic pelvic pain (CPP) after GnRH-a, Morelli et al. conducted a retrospective study on 63 premenopausal women with adenomyosis or endometriosis (28). The results showed that compared to baseline before treatment, a significant decrease in CPP intensity was observed in both adenomyosis group (*n* = 15) and endometriosis group (*n* = 48) (*p* < 0.05). This trend of reduction was more obvious in the adenomyosis group (*p* < 0.001). Meanwhile, a significant reduction was observed in days requiring analgesics (*p* < 0.01). GnRH-a can effectively relieve pain in patients with adenomyosis.

In fact, since adenomyosis is an estrogen-dependent disease, the hormonal environment of the patients will not be changed after HIFU treatment. Under estrogen stimulation, the lesions in the uterus may still recur or newly develop. HIFU combined with GnRH-a can help to maintain the efficacy of HIFU and reduce the recurrent rate. Our analysis showed that patients in the HIFU combined with GnRH-a group had a more pronounced decrease in uterine volume and lesion volume, and a lower recurrence rate. Most included studies suggested that patients should be injected GnRH-a three times after HIFU ablation. The first GnRH-a was administered on the first to third day of the first menstruation after HIFU treatment. Then, the interval between the two GnRH-a injections was 28 days.

Severe dysmenorrhea and excessive menstruation are the main symptoms that affect patients. Previous studies showed that the degree of adenomyosis infiltration of the uterus is related to the patient's pain (29). Gordts et al. proposed that hypermotility and increased expression levels of oxytocin receptors may be related to the degree of dysmenorrhea (30). Levgur et al. proposed that menorrhagia is related to the depth of adenomyotic lesion in the myometrium (31). A long-term results from single center proposed that HIFU had poor long-term efficacy for adenomyosis (32). The results of this retrospective analysis showed a significant effect in decreasing the dysmenorrhea score and the menorrhagia score at each follow-up time point. However, the effective rate of HIFU in alleviating dysmenorrhea and menorrhagia gradually decreased with the extension of follow-up time.

The results of our study showed that the symptoms of the two groups were all improved after the procedure, but the VAS scores for dysmenorrhea and menstrual volume scores of the HIFU combined with GnRH-a group were lower than those of the HIFU alone group. The serum CA125 levels was also decreased. Although the result of VAS scores for dysmenorrhea showed that HIFU combined with GnRH-a can better alleviate dysmenorrhea of patients, there still exists the excessive heterogeneity (*I*^2^ = 83%) between studies. Therefore, we performed a sensitivity analysis by removing each single study sequentially. When the study of TAN 2019 was omitted, the heterogeneity is significantly reduced (*I*^2^ = 65%). Figure 6 displayed that there is no significance (MD 0.15, 95% CI −0.47–0.77) between the experimental group and the control group in the study of TAN 2019. However, there still exists the heterogeneity. The low quality of included studies may cause it. Most studies used scale of Uterine Symptom and Quality of Life (UFS-QoL) to evaluate the patients' severity of symptoms and health-related quality of life (HRQOL) (23, 33). The symptom severity scores higher, the symptoms of patients worse, whereas the HRQOL scores higher, the HRQOL of patients better. Regrettably, none of the included studies systematically assessed these specific symptoms, and were lack of investigations and evaluations on patients' health-related quality of life (34).

The relationship between adenomyosis and infertility is unclear, but adenomyosis can adversely affect female fertility (35, 36). This is mainly related to the disruption and thickening of the myometrial junctional zone (JZ), and endometrial hypoacceptability (37). In recent years, owing to the continuous improvement of various ultrasound diagnostic methods and the increasing age of women seeking fertility treatment, the proportion of women with diagnosed adenomyosis among infertile women has increased. Traditionally, infertile patients with adenomyosis are treated by taking GnRH-a or surgically removing the adenomyotic lesion. Studies have pointed out that HIFU is a safe and effective procedure for infertile women and does not increase obstetric risks (38). Huang et al. performed a retrospective analysis on 93 patients with adenomyosis and infertility underwent USgHIFU (*n* = 50) or laparoscopic excision (LE) (*n* = 43) (39). They found that the pregnancy rate of the HIFU group (52%, 26/50) was significantly higher than that of the LE group (30.2%, 13/43). However, there is still a lack of high-quality randomized controlled trials comparing HIFU with other therapies. A meta-analysis revealed that infertile women receiving long-term GnRH-a treatment before receiving *in vitro* fertilization are associated with increased pregnancy rates (40). In this meta-analysis, only one study reported pregnancy outcomes. However, the sample size was too small, so we could not determine the group that could benefit more in terms of increased pregnancy rate. We look forward to more large-scale controlled clinical trials in the future.

Moreover, five studies reported adverse reactions after treatment (11, 13, 14, 17, 19), of which only two (17, 19) completely and uniformly reported specific data of the type and number of adverse reactions in the two groups. The results of the two articles indicated no statistically significant difference in the incidence of adverse reactions between the experimental and control groups (*P* > 0.05). In addition, three studies (11, 13, 14) also reported that no significant difference in the incidence of adverse reactions between the two groups. However, the three papers did not provide specific data. The combination of the number of adverse reactions in the two groups was not conducive for the comparison between the groups. In fact, owing to the working mechanism of the thermal effect of HIFU (41), patients in both the experimental and control groups had certain adverse reactions after HIFU ablation. We summarized and counted all the adverse reactions and the number of cases in the five articles (Table 2). Adverse reactions are mainly manifested as postoperative pain in the treatment area, sacral tail pain, and skin burning. According to the *Society of Interventional Radiology Clinical Practice Guidelines* (42) for the grade classification of adverse reactions, grades A and B are minor complications, and no serious complications occurred. The patients in the experimental group who took GnRH-a had symptoms such as amenorrhea and mood changes, hot flashes, night sweats, and insomnia. These symptoms will improve spontaneously at 2 months after discontinuation of the drug (14, 19), and no special treatment is required.

**Table 2:** Adverse reactions occurred in the patients after treatment.

	**Complications**	**Total (*n*)**	**SIR grade**
HIFU	Pain in treated region	62	A
	Low abdominal pain	15	A
	Sacral tail pain	37	A
	Radiating pain in the leg	10	A
	Neural response	4	A
	Skin burn	24	A
	Urinary retention	6	B
	Vaginal bleed discharge	26	A
	Thrombocytopenia	2	A
	Acute pelvic inflammatory disease	1	B
GnRH-a	Amenorrhea	58	A
	Mood change, Hot flashes, Night sweat, Insomnia	6	A

*Data expressed as n. HIFU, High Intensity Focused Ultrasound. GnRH-a, Gonadotropin-releasing hormone agonist. SIR, Society of Interventional Radiology clinical practice guidelines*.

This systematic review and meta-analysis has some limitations. First, all the studies were conducted in China and most of them were written in Chinese, with a certain risk bias. In fact, China has the independent intellectual property rights (IPR) for HIFU technology. Therefore, many studies related to HIFU is conducted in China or written in Chinese. And the qualities of some included studies were generally low. Information on the main outcomes was incomplete, and the allocation concealment and blinding methods were not mentioned in detail. Second, the sample sizes of some articles were small. Third, most studies did not report the specific volumes of the uterine and adenomyotic lesion after treatment but, instead, replaced it with the reduction rates of the uterine and adenomyotic lesion volumes, so the outcome indicators must be carefully referenced. Forth, some articles were lacked of long-term follow-up results and assessment of patient quality of life.

At present, HIFU with GnRH-a therapy has not been reported in terms of its effect on the improvement of patient quality of life and the increase in pregnancy rate. This may become a new direction for future research. This systematic review did not discuss the classification of adenomyosis. Whether this therapy has a difference in efficacy between focal and diffuse adenomyosis is still unknown, so more randomized controlled trials must be conducted.

## Conclusions

The results of this meta-analysis showed compared with HIFU treatment alone, HIFU with GnRH-a for the treatment of adenomyosis has greater efficacy in decreasing the volumes of the uterine and adenomyotic lesions and alleviating symptoms. However, since the number of the included studies was too small and most of them were written in Chinese, this conclusion needs to be referenced with caution. The long-term evidence of its efficacy is still insufficient.

## Data Availability Statement

The original contributions generated for the study are included in the article/supplementary material, further inquiries can be directed to the corresponding author.

## Author Contributions

L-LP developed the search strategy and completed the manuscript writing. L-LP and JM completed the electronic search and selected appropriate literature. R-NL and T-TZ extracted the information of the selected studies. L-XF was responsible for data analysis using RevMan 5.3 software. YW advised on the data analysis and was responsible for correspondence. All authors carefully checked and approved the final manuscript.

## Conflict of Interest

The authors declare that the research was conducted in the absence of any commercial or financial relationships that could be construed as a potential conflict of interest.

## Publisher's Note

All claims expressed in this article are solely those of the authors and do not necessarily represent those of their affiliated organizations, or those of the publisher, the editors and the reviewers. Any product that may be evaluated in this article, or claim that may be made by its manufacturer, is not guaranteed or endorsed by the publisher.
